# EVERYDAY SOLITUDE, TIME SAVORING, WELL-BEING, AND HEALTH IN OLDER ADULTHOOD

**DOI:** 10.1093/geroni/igad104.1819

**Published:** 2023-12-21

**Authors:** Theresa Pauly, Miriam Wallimann, Shira Peleg

**Affiliations:** Simon Fraser University, Vancouver, British Columbia, Canada; University of Zurich, Zurich, Zurich, Switzerland; Simon Fraser University, Vancouver, British Columbia, Canada

## Abstract

Despite the significance of social relationships, solitude (being alone and without social interaction) forms an essential part of older adults’ lives. The current study aimed to examine the role of time savouring (i.e., the ability to attend to positive experiences and up-regulate positive feelings) for mitigating associations of solitude with indicators of negative well-being and health in old age. For this purpose, a sample of 108 older adults aged 65–92 years took part in a daily diary study. Over 14 days, participants reported daily amount of time spent in solitude, extent of time savouring, levels of depressive mood, and somatic symptoms in an end-of-day diary. Data were analysed using multi-level models controlling for study day, age, gender, education, and living alone. Participants reported higher depressive mood but not more somatic symptoms on days on which they spent more time in solitude than usual. On days on which participants indicated higher time savouring than usual, they reported lower depressive mood and lower levels of somatic symptoms. Importantly, associations of solitude with depressive mood and somatic symptoms were weaker on days with increased time savouring. There were no person-level associations between solitude or time savouring with depressive mood or somatic symptoms. The study’s findings highlight the importance of everyday time savouring in attenuating negative health-related aspects of solitude in older adults. Preserving and promoting time savouring in older adulthood may thus promote health and well-being in this age group.

